# Potent Inhibition of *Pseudogymnoascus destructans*, the Causative Agent of White-Nose Syndrome in Bats, by Cold-Pressed, Terpeneless, Valencia Orange Oil

**DOI:** 10.1371/journal.pone.0148473

**Published:** 2016-02-05

**Authors:** Nicholas Boire, Sean Zhang, Joshua Khuvis, Rick Lee, Jennifer Rivers, Philip Crandall, M. Kevin Keel, Nicole Parrish

**Affiliations:** 1 The Johns Hopkins School of Medicine, Baltimore, MD, United States of America; 2 The Johns Hopkins Bloomberg School of Public Health, Baltimore, MD, United States of America; 3 The University of Arkansas, Fayetteville, AR, United States of America; 4 UC Davis School of Veterinary Medicine, Davis, CA, United States of America; CSIRO, AUSTRALIA

## Abstract

The causative agent of White-nose Syndrome (WNS), *Pseudogymnoascus destructans*, has been shown to be fatal to several species of bats in North America. To date, no compounds or chemical control measures have been developed which eliminates the growth of the fungus in the environment or in affected animals. In the current study, we evaluated the activity of cold-pressed, terpeneless orange oil (CPT) against multiple isolates of *P*. *destructans in vitro*. For all assays, a modified Kirby-Bauer disk diffusion assay was used. Standardized spore suspensions were prepared, adjusted to a specific optical density, and used to plate fungal lawns. Plates were incubated at either 15°C or 4°C for up to 6 months and checked at regular intervals for growth. Once controls had grown, zones of inhibition were measured (mm) on test plates and compared to those obtained using current antifungal drugs. All *P*. *destructans* isolates were completely inhibited by 100% CPT (10 μL) at 1 month of incubation regardless of temperature (4°C and 15°C). Complete inhibition persisted up to 6 months following a single exposure at this concentration. Of the standard antifungals, only amphotericin B demonstrated any activity, resulting in zone diameters ranging from 58 mm to 74 mm. CPT, at the highest concentration tested (100%), had no significant effect against a variety of other environmental organisms including various filamentous fungi, bacteria and aerobic actinomycetes. Given that CPT is relatively non-toxic, the possibility exists that the all-natural, mixture could be used as an environmental pre-treatment to eradicate *P*. *destructans* from bat habitats. Additional studies are needed to assess any undesirable effects of CPT on bat behavior and health and overall impacts on other members of the interconnected ecosystem(s).

## Introduction

White-nose syndrome (WNS) is a lethal disease in bats caused by a psychrophilic (cold-adapted) fungus, *Pseudogymnoascus destructans* (synonym: *Geomyces destructans)*. To date, this fungus has killed an estimated 7 million bats in North America since first being identified from a solitary New York cave system in 2006 [1–8]. At the time of this writing, *P*. *destructans* has been identified in 29 states and 5 Canadian provinces [9]. Bat populations have been decimated and are continuing to rapidly decline due to a mortality rate of nearly 100 percent [1, 5, 6, 7, 10, 11]. WNS currently affects seven species of bats, with at least two officially labeled as endangered, including the Indiana bat (*Myotis sodalis*) [1, 5–7]. The infection causes the premature emergence of bats from their hibernation cycle, forcing them to survive in winter in the absence of their traditional food supply being available. Afflicted bats often appear emaciated exhibiting significant dehydration, with external epidermal lesions and white fungal growth appearing on their snouts [12–15]. Due to the current and potentially devastating ecological impact and severity posed by this pathogen, organizations from every level of government and private environmental conservation groups have come together to develop strategies for controlling WNS infection and spread, including the U.S. Fish and Wildlife Service, the National Park Service, the Department of Agriculture, and Bat Conservation International to name a select few [16, 17]. Recently, investigators demonstrated potential biological control of *P*. *destructans* by *Rhodococcus rhodochrous* which produced contact-independent activity against this wildlife pathogen [18]. However, no compounds or chemical treatments are currently described which successfully inhibit or control the growth of this fungus, either in the environment, or on the bats. In the current study, we evaluated the activity of CPT against *P*. *destructans* isolates recovered from bats in diverse geographic locations within the continental US and a number of other commonly encountered environmental organisms including various filamentous fungi, bacteria, and aerobic actinomycetes.

## Materials and Methods

**Fig 1:**
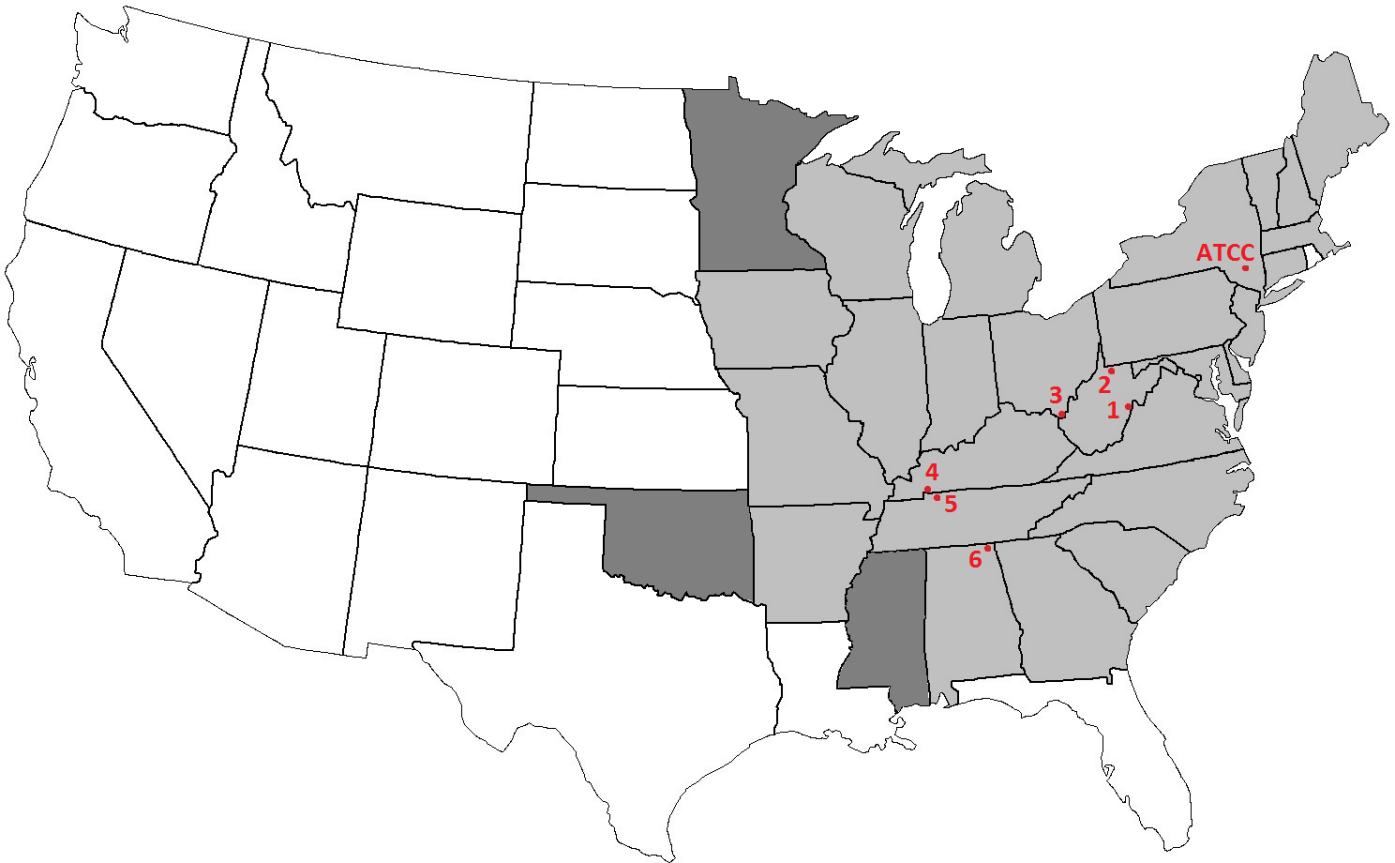
The continental United States, highlighting sites from which the *P*. *destructans* isolates were obtained for the current study and current states with confirmed cases of White-nose Syndrome. Isolate #1: Pocahontas County, West Virginia. Isolate #2: Monongalia County, West Virginia. Isolate #3: Lawrence County, Ohio. Isolate #4: Trigg County, Kentucky. Isolate #5: Montgomery County, Tennessee. Isolate #6: Jackson County, Alabama. The ATCC type isolate (MYA-4855) was previously recovered from Ulster County, New York. Lightly shaded states represent those with confirmed cases of White-nose Syndrome in bats caused by *P*. *destructans*; darker shaded states represent those most recently confirmed for positive identification of the fungus.

A modified version of the Kirby-Bauer disk diffusion assay was used to determine the *in vitro* susceptibility of several isolates *of P*. *destructans* to CPT, a commercially available mixture prepared from *Citrus sinensis* by Firmenich Citrus Center, Lakeland, FL. In total, 4 different lots were tested: two recently manufactured (< 1 month old), one ~5 years of age, and one > 7 years. Assessment of potency was done using 2-fold dilutions of each parent CPT mixture in DMSO beginning with 100% down to 0.19%. All dilution assays included a separate DMSO control. For all assays, the type isolate of *P*. *destructans* (American Type Culture Collection, ATCC MYA-4855) and 6 wild isolates (Research Archive Collection of Dr. Kevin Keel) were used, the latter having been obtained from diverse geographic locations as illustrated in Fig 1. All *P*. *destructans* isolates were cultured on Sabouraud Dextrose agar (Becton Dickinson, Sparks, Maryland) for one week at 15°C to permit adequate sporulation for preparation of spore suspensions in sterile water. To decrease clumping, suspensions were vortexed with sterile glass beads (1mm, Sigma-Aldrich, St. Louis, Missouri) for one minute and allowed to settle for an additional ten minutes after which the supernatant was adjusted to an optical density of 0.09–0.13 at 530 nm using a T20 spectrophotometer (Spectronic 20D+, Thermo Scientific, Waltham, MA). Adjusted suspensions were used to plate fungal lawns for each isolate tested to which were added sterile, blank filter disks (6 mm, Becton Dickinson). For all assays, 10 μL of CPT, ranging from 100% to 0.19%, were pipetted onto each blank disk and allowed to air dry in a biological safety cabinet for 10 minutes. For *P*. *destructans*, plates were incubated at 15°C and 4°C and examined daily for growth for the first 2 months and then weekly thereafter for a total of 6 months. To control for CPT inhibition sensitive to differences in temperature and growth rate, both were monitored in untreated controls to determine the number of days required at 15°C and 4°C for the appearance of fungal colonies. Once confluent growth was observed on control plates, the zone of inhibition was measured in mm for CPT and all other anti-fungal drugs tested. Zones obtained with CPT were compared to those resulting from standard antifungal drugs: amphotericin B (10 μg, Rosco Diagnostics, Taastrup, Denmark), caspofungin, (5 μg, Rosco Diagnostics), fluconazole (25 μg, Becton Dickinson), and voriconazole (1 μg, Becton Dickinson). These drugs were used as a comparative reference for CPT-mediated zones of inhibition to establish relative susceptibility or resistance since no current interpretive guidelines exist for this natural antimicrobial. Interpretation of zone diameters for the standard antifungals was made using currently established breakpoints and recommendations as per the Clinical and Laboratory Standards Institute [19]. CPT was also tested against a variety of other environmental filamentous fungi, bacteria and aerobic actinomycetes from the Johns Hopkins Hospital research archive including: *Aspergillus niger*, *A*. *terreus*, and *A*. *fumigatus*, *Scedosporium prolificans*, *Paecilomyces variotii*, *Fusarium solani*, *Bacillus* spp., *Acinetobacter baumanii*, *Stenotrophomonas maltophilia*, *Nocardia* spp., *Rhodococcus* spp., and *Mycobacterium fortuitum* and *M*. *phlei*. For these additional environmental organisms, inoculum preparation, incubation times, and conditions varied. For all filamentous fungi, cultures and spore suspensions were prepared as above for *P*. *destructans;* plates were incubated at 25°C and examined daily for growth for up to 1 week. For Gram-positive and Gram–negative bacteria, and *Rhodococcus* spp., all suspensions were prepared from 24 hour growth on solid media (Mueller-Hinton agar plates, Becton Dickinson) and adjusted to a 0.5 McFarland turbidity standard in Mueller-Hinton broth (Becton Dickinson). Subsequently, lawns were plated onto Mueller-Hinton agar plates followed by the addition of blank, sterile disks and varying concentrations of CPT as described above. Plates were incubated at 35°C and CPT-mediated zone diameters measured after 24 hours of incubation for all bacteria; 3 days for *Rhodococcus* spp. For *Nocardia* spp. and *Mycobacteria*, suspensions were prepared from growth on Middlebrook 7H10 agar (HiMedia Labs, VWR, Radnor, PA) and adjusted to a 0.5 McFarland turbidity standard using Middlebrook 7H9 broth (Hardy Diagnostics, Santa Maria, CA). Subsequently, lawns were plated onto organism-specific agar plates (bacteria, Mueller-Hinton agar; *Nocardia* and *Mycobacteria*, Middlebrook 7H10 agar) and the plates incubated at 25°C for all filamentous fungi, and 35°C for all bacteria, *Nocardia*, and *Mycobacteria*. Bacterial and filamentous fungal plates were read at 24 hours; Nocardia and mycobacterial plates were read at 5 days and 7 days, respectively. All assays were performed in triplicate.

## Results

**Fig 2:**
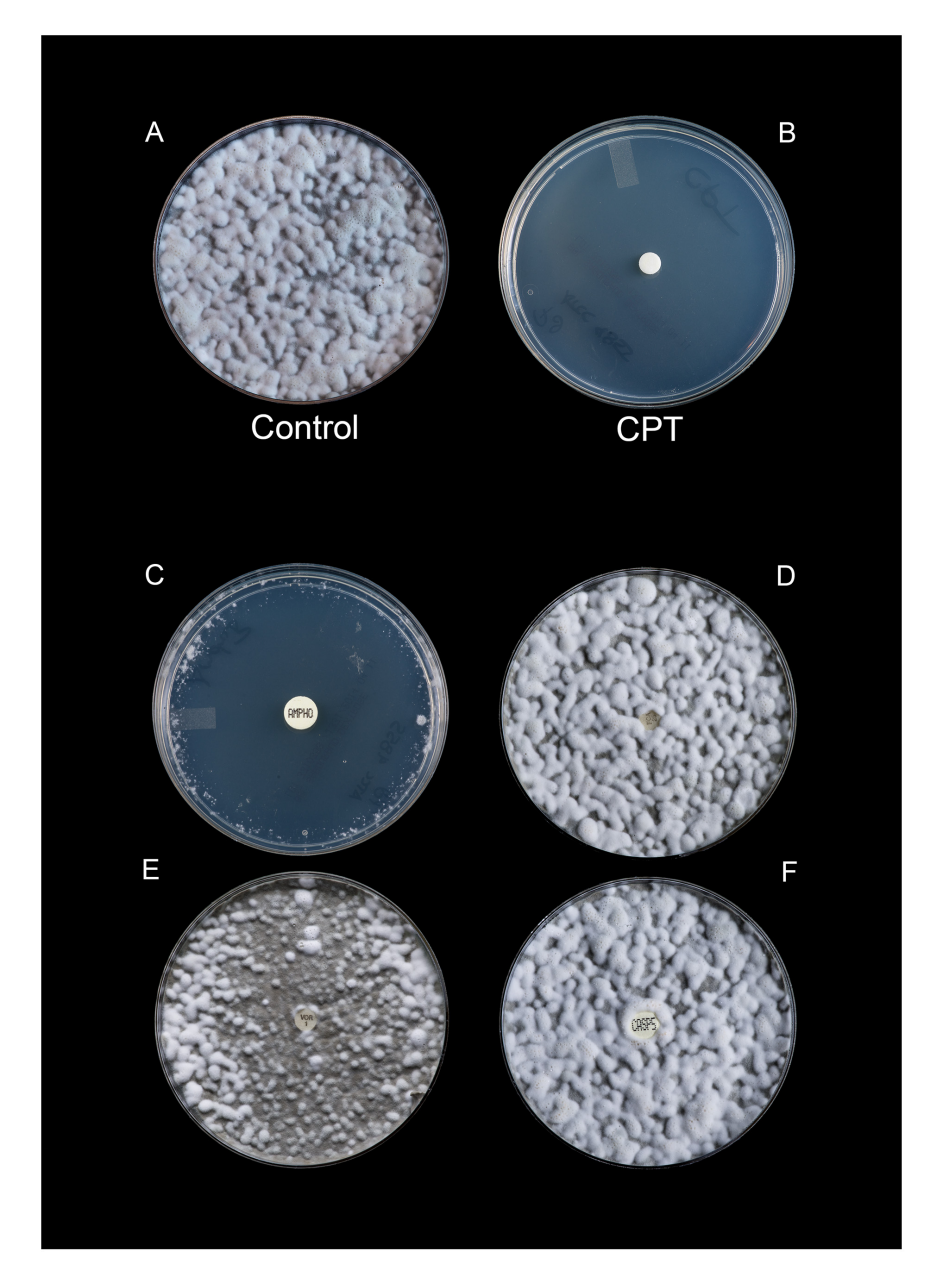
Inhibitory effect of cold-pressed, terpeneless Valencia orange oil against the type isolate (ATCC MYA-4855) of *P*. *destructans* after 6 months incubation at 4°*C*. A) untreated control; B, 100% CPT (10 μL); C) amphotericin B (10 μg/mL); D, fluconazole (25 μg/mL); E, voriconazole (1 μg/mL); and F, caspofungin (5 μg/mL).

**Fig 3:**
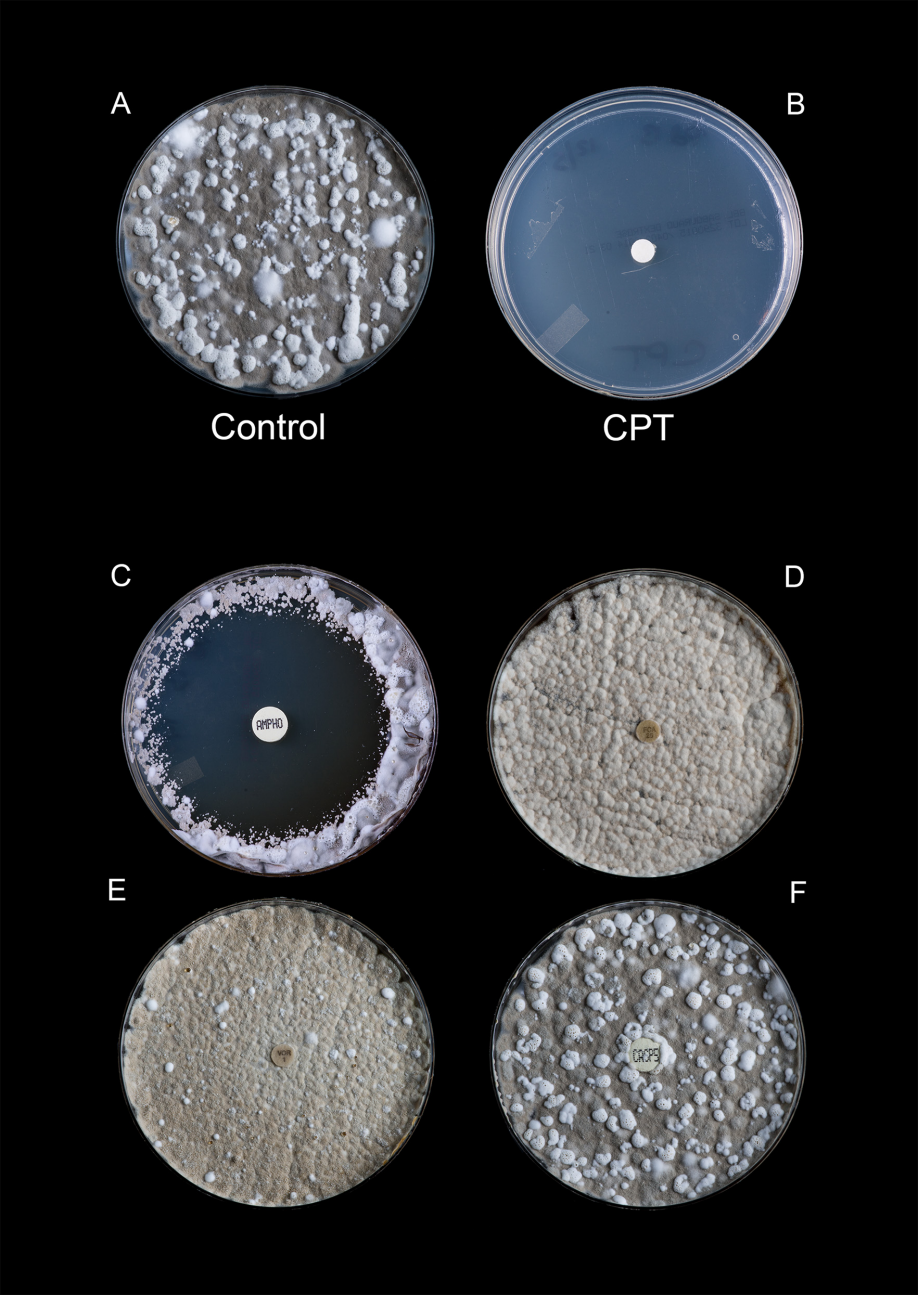
Inhibitory effect of cold-pressed, terpeneless Valencia orange oil against a wild isolate of *P*. *destructans* (*Pd* #13) after 6 months incubation at 4°*C*. A) untreated control; B, 100% CPT (10 μL); C) amphotericin B (10 μg/mL); D, fluconazole (25 μg/mL); E, voriconazole (1 μg/mL); and F, caspofungin (5 μg/mL).

The difference in growth rate between the cold-adapted *P*. *destructans* isolates under the two temperatures studied was negligible. At 15°C, isolates grew in a complete lawn in an average of 21 days (range 18–24 days) and at 4°C isolates demonstrated growth in an average of 28 days (range 25–31 days). All *P*. *destructans* isolates tested were completely inhibited by 10 μL of 100% CPT at 1 month of incubation at both 15°C and 4°C. Complete inhibition continued with this concentration of CPT (lots ≤ 5 years of age) for up to 6 months or the duration of the assay (Figs 2 and 3). For the CPT lot > 7 years of age, complete inhibition at the same concentration began to wane by the end of the second month of incubation with an average zone size of 60 mm (range 45 mm to 72 mm); by month 4, the plates were completely overgrown. When diluted to < 100%, the lots of CPT < 1 month of age demonstrated appreciable inhibitory activity at 3 months of incubation with average zone diameters ranging from 40 mm (range: 37 mm to 46 mm; 50% CPT), to 18 mm (range: 6 mm to 13 mm; 6.25% CPT) (Table 1). No inhibition was seen at concentrations < 6.25% (Table 1). In contrast, the two older lots of CPT showed little to no inhibition of growth at any concentrations tested below 100% with average zone diameters at 3 months of incubation ranging from 15 mm (50% CPT) to 8 mm (6.25% CPT); no inhibition was demonstrated at concentrations < 6.25%. No inhibition was observed in any of the assays with the diluent (DMSO) alone. Of the standard antifungal agents tested, only amphotericin B demonstrated any inhibitory activity against any of the *P*. *destructans* isolates tested over the course of the assay. However, zone diameters did vary between isolates (15°C, 42 days: range 58-65mm; 4°C, 35 days: range 65–74 mm). Caspofungin, voriconazole, and fluconazole failed to inhibit any *P*. *destructans* isolates regardless of time-point or incubation temperature (Figs 2 and 3). For the other environmental filamentous fungi, bacteria, and aerobic actinomycetes tested, CPT had little to no effect with zone diameters ≤ 15 mm following exposure to 10 μL of 100% CPT.

**Table 1:** Inhibitory effect of various % concentrations of CPT (< 1 month of age) against isolates of *P*. *destructans* at 3 months of incubation.

Isolate	%CPT (Zone diameters in mm^a^)					
	100%	50%	25%	12.5%	6.25%	≤ 3.0%
**ATCC (MYA-4855)**	80^b^ ± 0.0	42 ± 2.5	26 ± 2.6	19 ± 1.2	11 ± 0.9	6^c^ ± 0.0
***Pd*04**	80 ± 0.0	43 ± 3.2	22 ± 3.1	15 ± 1.2	6 ± 0.0	6 ± 0.0
***Pd*13**	80 ± 0.0	37 ± 7.5	30 ± 3.7	18 ± 1.6	6 ± 0.0	6 ± 0.0
***Pd*17**	80 ± 0.0	39 ± 2.5	21 ± 2.9	8 ± 1.2	12 ± 1.6	6 ± 0.0
***Pd*21**	80 ± 0.0	46 ± 6.0	15 ± 0.5	19 ± 1.2	13 ± 1.9	6 ± 0.0
***Pd*39**	80 ± 0.0	37 ± 6.2	12 ± 1.6	16 ± 2.1	11 ± 1.9	6 ± 0.0
***Pd*52**	80 ± 0.0	38 ± 6.7	18 ± 2.4	16 ± 3.0	12 ± 1.7	6 ± 0.0

^a^Inhibition zones (mm) are average values of three separate assays ± the standard deviation of the mean.

^b^A zone diameter of 80 mm = complete inhibition, no growth visualized on the entire plate.

^c^A zone diameter of 6 mm = no inhibition seen, a confluent lawn visible; 6 mm is equivalent to the diameter of the disk.

## Discussion

Since its discovery in 2006, WNS has killed millions of bats throughout North America. The effects of this devastating pathogen are immense, and may cause profound environmental, economic, agricultural, and public health impacts. Bats play a critical role in their respective ecosystems. Insectivorous bats consume large amounts of insects with some eating the equivalent of half their body weight per night [20]. Increased insect populations may result in significant agricultural losses and costs due to the requirement for more pesticides as well as detrimental effects on public health as insect vectors of infectious diseases could be expected to increase. Current estimates postulate the possible damage to agriculture to range from a low of $3 billion to a high of $53 billion per year [21]. Still to be determined are the ramifications to public health [21]. Thus, it is vital that efforts continue to discover a means to combat and control this deadly fungus.

In the current study, exposure of all isolates of *P*. *destructans* to 100% CPT (10 μL) ≤ 5 years of age resulted in complete inhibition of fungal growth up to 6 months of incubation following a single exposure regardless of temperature (15°C or 4°C). At concentrations < 100%, only the CPT lots < 1 month of age demonstrated any activity with no inhibition observed at concentrations < 6.25% at the same time-point. Taken together, this suggests that CPT is relatively stable at room temperature for up to 5 years with no significant loss in potency when used full strength (100%). However, when diluted below 100%, only the lots < 1 month of age maintained significant anti-*P*. *destructans* activity, indicating that the older lots have lost potency over time, especially with regard to the lot > 7 years of age. This is not unexpected as even under refrigerated storage conditions, many of the compounds in the parent CPT mixture would be expected to oxidize over time. Importantly, none of the commercially available antifungals used as a comparator in this study, showed similar activity *in vitro* at the same time-point, including fluconazole and voriconazole, which had been shown previously to be inhibitory to *P*. *destructans* [22]. This discrepancy may be related to methodological differences in the assays performed: E-test and microbroth dilution used in the earlier investigation versus the modified Kirby-Bauer method employed in the current study. Additionally, the time-points at which assays were read and interpreted varied between the two investigations. For example, in the prior study, endpoints were read and interpreted after 10 to 14 days of incubation at 15°C; after 21 days at 6°C. In the current study, plates were read and interpreted following a minimum of 21 days, extending out to 6 months which may explain the decreased inhibition of fluconazole and voriconazole as observed in this investigation. In addition, dissimilarities in the *in vitro* activity of CPT versus the other drugs used in this study may also be due to potential differences in both the mechanism of action of CPT versus the current antifungals as well as the relative concentrations used of each. The specific mechanism of action of amphotericin B, fluconazole, voriconazole and caspofungin have been well defined, however, determination of the mechanism of CPT-mediated inhibition of *P*. *destructans* was beyond the scope of this current project. Other investigators have previously speculated and or demonstrated that CPT affects several key cellular processes such as membrane maintenance and integrity as well as ATP production and metabolism in target organisms [23–25]. For instance, transcriptional profiling in *Staphylococcus aureus* exposed to CPT revealed changes in cell wall-associated genes leading the authors to conclude that the primary effect of CPT was on the cell wall [23]. In mycobacteria, including *M*. *tuberculosis* and *M*. *bovis* BCG, CPT-specific effects were noted in ATP synthesis and associated downstream energy-dependent metabolic processes such as mycolic acid production [24]. Similar studies demonstrated membrane-active effects from essential oils in various microorganisms resulting in disruption of membrane integrity and or permeability [26–28]. Of the commercially available antifungals used for comparison purposes in this study, only amphotericin B, which binds to ergosterol, showed any demonstrable activity *in vitro*. None of the remaining antifungals, which inhibit ergosterol or glucan synthesis, had any activity against *P*. *destructans*. Taken together, this suggests that the CPT-mediated inhibition of *P*. *destructans*, may be due to a mechanism of action which differs from that of current antifungal drugs.

Unlike antibiotics, essential oils, including CPT, are complex mixtures of compounds, some of which are volatile. Most are ‘generally regarded as safe’ (GRAS) by the FDA and are frequently used as flavoring agents in foods, cosmetics, and also cleaning products. In fact, some oils are currently being investigated as potential alternatives to conventional pesticides due to their low toxicity and a more favorable impact on the environment [29]. Although the specific effect of CPT on bats is not known, it has been shown to be non-toxic to mammalian keratinocytes at concentrations sufficient to eliminate *S*. *aureus*, prompting the authors to suggest its use as a topical antimicrobial against this specific pathogen [30]. Additional testing is needed to determine if CPT is equally non-toxic to bats.

In summary, these results demonstrate that CPT-mediated inhibition of *P*. *destructans* is possible *in vitro* and persists up to 6 months at optimal incubation temperatures required for growth following a single application. As such, CPT may provide a novel chemical means to help control this deadly fungus in the environment, without disturbing beneficial bacteria such as *R*. *rhodochrous*, which has been shown to have contact-independent activity against *P*. *destructans*. Significant research is still needed to determine if any of the volatile components of CPT are sufficient for killing *P*. *destructans*, the effect(s) on bat behavior and physiology, as well as the impact on related ecosystems to avoid any undesirable effects.
